# Finite element analysis of long proximal femoral bionic nail (PFBN) fixation for femoral shaft fractures combined with ipsilateral femoral neck fractures

**DOI:** 10.3389/fbioe.2026.1799331

**Published:** 2026-04-22

**Authors:** Zixi Fan, Kai Ding, Wei Liu, Biyou Luo, Tailong Shi, Yuqin Zhang, Qi Zhang, Wei Chen

**Affiliations:** 1 Department of Orthopaedic Surgery, Hebei Medical University Third Hospital, Shijiazhuang, Hebei, China; 2 Engineering Research Center of Orthopedic Minimally Invasive Intelligent Equipment, China Ministry of Education, Shijiazhuang, Hebei, China; 3 Key Laboratory of Biomechanics of Hebei Province, Shijiazhuang, Hebei, China; 4 NHC Key Laboratory of Intelligent Orthopaedic Equipment, Shijiazhuang, Hebei, China; 5 Pharmacy Department, Cangzhou Maternal and Child Health Hospital, Cangzhou, China; 6 Department of Cardiology, The Third Hospital of Hebei Medical University, Shijiazhuang, China

**Keywords:** combined approach, finite element analysis, integrated structure, ipsilateral femoral neck and shaft fractures, PFBN

## Abstract

**Objective:**

Femoral reconstruction nails (RN) and three cannulated screws combined with a plate (TCP) are commonly employed for the treatment of ipsilateral femoral neck and shaft fractures (IFNSF). However, these fixation strategies are associated with a considerable incidence of postoperative complications, reflecting the biomechanical challenges of this fracture pattern. Therefore, this study aims to compare the biomechanical performance of a novel long proximal femoral bionic nail (PFBN) with these conventional fixation methods for the treatment of this complex fracture pattern.

**Methods:**

Computed tomography (CT) data of the femur were obtained from a healthy adult male volunteer to reconstruct a three-dimensional femoral model using Mimics 21.0 and Geomagic Studio 2013. A combined femoral shaft and ipsilateral femoral neck fracture model was then established in UG 12.0. Three internal fixation constructs—the long PFBN, RN, and TCP—were designed and assembled to simulate surgical fixation. Finite element analysis was subsequently performed using Abaqus 2022 to evaluate stress distribution and displacement characteristics among the three fixation constructs.

**Results:**

Under the three loading conditions, the long PFBN exhibited lower or comparable stress and displacement within the fixation constructs. Compared with the TCP construct, the long PFBN showed a more uniform stress distribution. In most loading scenarios, its mechanical performance was comparable to, or slightly better than, that of the RN construct. Analysis of fracture-site micromotion indicated that tangential micromotion at the femoral neck fracture interface in the long PFBN construct was intermediate between that of the RN and TCP constructs.

**Conclusion:**

In this finite element analysis, the long PFBN demonstrated relatively favorable biomechanical performance under axial, bending, and torsional loading conditions. These findings may provide preliminary biomechanical insights into the comparative performance of different fixation strategies for this complex fracture pattern.

## Introduction

With the rapid development of transportation and construction industries, the incidence of ipsilateral femoral neck and shaft fractures (IFNSF) has increased in recent years, accounting for approximately 1%–9% of femoral shaft fractures (Mohan et al., 2019). The management of this complex injury remains challenging, with substantial variability in clinical outcomes. The overall complication rate is higher than that observed in isolated femoral shaft or femoral neck fractures. Reported complications include coxa vara (up to 28%) (Tsai et al., 2009), infection (up to 10%) (Du et al., 2016), malunion (up to 7%) (Tsai et al., 2009), nonunion (5%–9% for femoral shaft and 1%–2% for femoral neck) (Tsai et al., 2009; Ostrum et al., 2014), avascular necrosis of the femoral head (up to 4%) (Tsai et al., 2009), and internal fixation failure.

Currently, the clinical management of this fracture type primarily relies on two strategies: femoral reconstruction nails (RN) and cannulated screws combined with a plate (TCP) (Singh et al., 2008). Each approach offers distinct advantages. The RN provides intramedullary central fixation, with a load-bearing axis closer to the mechanical axis of the lower limb, which may facilitate more uniform stress transmission and reduce disruption to the blood supply at the fracture site. In addition, both fractures can be addressed simultaneously using a single implant (Zhao et al., 2023; Dahuja et al., 2018). In contrast, the TCP strategy enables separate and relatively rigid fixation of the femoral neck and shaft, thereby providing more direct local control. However, clinical observations and previous studies suggest that both strategies are associated with certain biomechanical limitations. For proximal femoral fractures, both RN and TCP typically adopt parallel screw configurations, which have been associated with complications such as screw loosening, cut-out, loss of the neck–shaft angle, and avascular necrosis of the femoral head under complex loading conditions (Gadegone et al., 2017; Samsami et al., 2019; Bojan et al., 2018). For femoral shaft fractures, these fixation methods involve intramedullary central fixation (RN) and extramedullary eccentric fixation (TCP), respectively. In the TCP system, the screws and plate are not integrated into a single construct, which may lead to discontinuous load transfer. This may contribute to stress shielding at the fracture interface and potentially affect the bone healing process (Baghel et al., 2021; Angelini et al., 2021).

The occurrence of these complications is likely multifactorial; however, the mismatch between internal fixation devices and the anatomical and biomechanical environment of the femur may represent an important contributing factor. To improve internal fixation strategies for proximal femoral fractures, the research team led by Academician Yingze Zhang investigated the trabecular architecture of the proximal femur and proposed the concept of triangular-supported fixation (TSF). This concept involves constructing a stable triangular support structure to restore the physiological load distribution of tensile and compressive trabeculae in the proximal femur. Such a design may enhance fixation strength and improve rotational stability (Zhu et al., 2021; Zhang Kaixuan et al., 2024). Based on this concept, the proximal femoral bionic nail (PFBN) was developed. Previous finite element analyses and preliminary clinical studies suggest that PFBN exhibits favorable biomechanical performance in proximal femoral fractures, such as intertrochanteric fractures (Zhang Yi-Fan et al., 2024; Ding et al., 2023). However, the application of this concept and the long PFBN in the treatment of femoral shaft fractures combined with ipsilateral femoral neck fractures remains insufficiently investigated from a biomechanical perspective.

Therefore, this study aimed to compare the biomechanical performance of a long proximal femoral bionic nail (PFBN), based on the triangular-supported fixation concept, with two conventional internal fixation methods—femoral reconstruction nail (RN) and cannulated screws combined with a plate (TCP)—using a finite element model of IFNSF. Stress distribution, displacement patterns, and fracture-site micromotion were analyzed to evaluate the biomechanical performance of the long PFBN for this complex fracture pattern.

## Materials and methods

### General information

A healthy adult male volunteer (body weight: 73 kg) with no history of fractures, tumors, or limb trauma was included in this study. The study protocol was approved by the Medical Ethics Committee of the Third Hospital of Hebei Medical University (Approval No. Ke2021-059-1). The requirement for written informed consent was waived.

### Image data acquisition

A 64-slice spiral computed tomography (CT) scanner was used to acquire images of the lower limb of the volunteer. The scanning range extended from the hip joint to the knee joint to ensure complete femoral CT data acquisition. All images were stored in Digital Imaging and Communications in Medicine (DICOM) format for subsequent analysis.

### Femur model reconstruction

The DICOM files were imported into Mimics 21.0 software (Materialise, Belgium) for three-dimensional reconstruction. Based on the CT attenuation values of different tissues, grayscale threshold values were defined to segment the initial femoral structure. Region growing, mask editing, and smoothing procedures were applied to generate a geometric model of the femur, which was subsequently exported in STL format. The femoral geometry was then imported into Geomagic Studio 2013 (Geomagic, USA), where noise reduction, surface smoothing, feature removal, contour editing, mesh generation, and surface fitting were performed to reconstruct a solid femoral model. Subsequently, separate models of cortical and cancellous bone were established.

### Fracture and fixation model establishment

**FIGURE 1:**
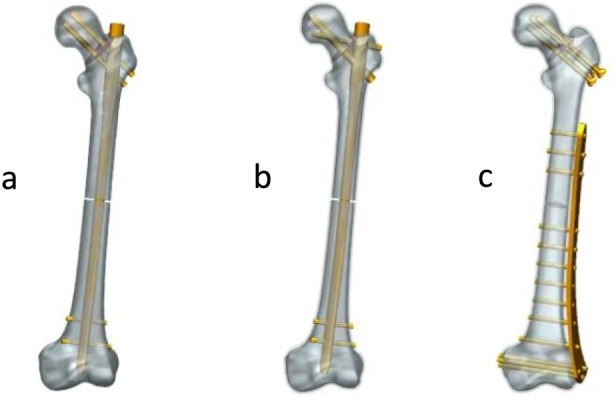
Schematic diagram of the three internal fixation devices and fracture models. **(a)** long PFBN **(b)** RN **(c)** TCP.

The geometries of the internal fixation devices were designed in UG 12.0 (Siemens PLM Software, Germany) according to manufacturer specifications. The intramedullary nails had a length of 380 mm, a diameter of 11 mm, and a neck–shaft angle of 125°. The distal locking screws had a diameter of 5 mm. For the PFBN, the proximal fixation screw and support screw had diameters of 7.5 mm and 5.5 mm, respectively. For the RN, the proximal screws had a diameter of 6.5 mm. The plate used in the TCP system had a length of 330 mm and a thickness of 5 mm, while the cannulated screws had a diameter of 6.5 mm and a length of 95 mm. Based on previous studies (Alborno et al., 2023; Wolinsky and Johnson, 1995; Alho, 1996), an unstable Pauwels type III femoral neck fracture was created at the base of the femoral neck. Additionally, approximately 2 mm of bone was removed from the midshaft to simulate a comminuted femoral shaft fracture (Shengyuan, 2022). The fracture models were then assembled with the corresponding fixation devices according to standard orthopedic surgical principles by an experienced orthopedic surgeon. To reduce computational cost, screw threads were simplified as smooth cylindrical surfaces. The assembled models were exported in STP format for subsequent analysis (Figure 1).

The complete femoral and fixation models were imported into HyperMesh 13.0 software (Altair, USA) for mesh generation. Both bone and implant models were meshed using C3D4 tetrahedral elements. Mesh convergence analysis demonstrated that the variation in key outcome parameters was less than 5%, indicating adequate mesh convergence. The mesh size of both cortical and cancellous bone was set to 1.5 mm. The femoral model consisted of 125,789 nodes and 496,822 elements. The numbers of nodes and elements for the three models are summarized in Table 1.

**TABLE 1:** The number of nodes and elements in the three models.

Components	Nodes	Elements
Long PFBN	187962	764162
RN	188656	769867
TCP	195506	779283

### Material properties and boundary conditions

The mesh models were imported into Abaqus 2022 (Dassault Systèmes, France) for finite element analysis. As shown in Table 2, and based on previous studies (Shengyuan, 2022), all bone tissues and internal fixation devices were assumed to be homogeneous, isotropic, and linearly elastic materials, and the corresponding material properties were assigned accordingly. According to previous studies (Anwar et al., 2020), the contact interactions and boundary conditions were defined as follows: the interface between cancellous and cortical bone was modeled as a tied contact; the fracture surfaces were defined as sliding contact with a friction coefficient of 0.46 (Hong et al., 2017). The friction coefficient between bone and implants was set to 0.3 (Chen et al., 2004), and that between implant components was set to 0.2 (Lee et al., 2016).

**TABLE 2:** Material parameters.

Part	Young’s modulus, E (GPa)	Poisson’s ratio, ν
Cortical bone	17	0.3
Cancellous bone	1.3	0.3
Titanium alloy	110	0.3

**FIGURE 2:**
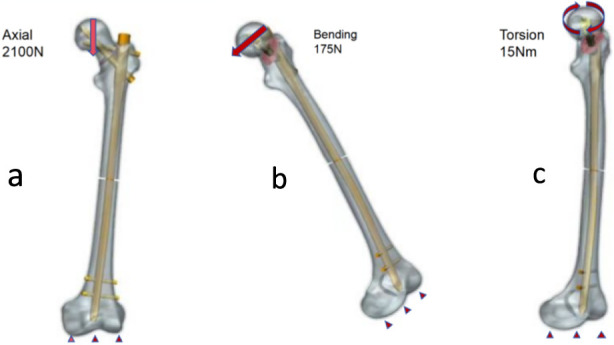
Boundary Conditions under axial **(a)**, bending **(b)**, and torsional **(c)** Loading.

As illustrated in Figure 2 and based on previous studies (Yang et al., 2025), three loading conditions were simulated: axial, bending, and torsional loading. The distal end of the femur was fully constrained by restricting all six degrees of freedom. Under axial loading, a uniformly distributed load equivalent to approximately three times the body weight was applied to the femoral head to simulate the peak load experienced by the hip joint during walking. A lateral load of 175 N was applied to represent forces acting on the femur during activities such as normal gait and lateral bending. A torsional moment of 15 Nm was applied to the femoral head to represent rotational loading experienced by the proximal femur during daily activities.

## Results

### Validation of the finite element model

**FIGURE 3:**
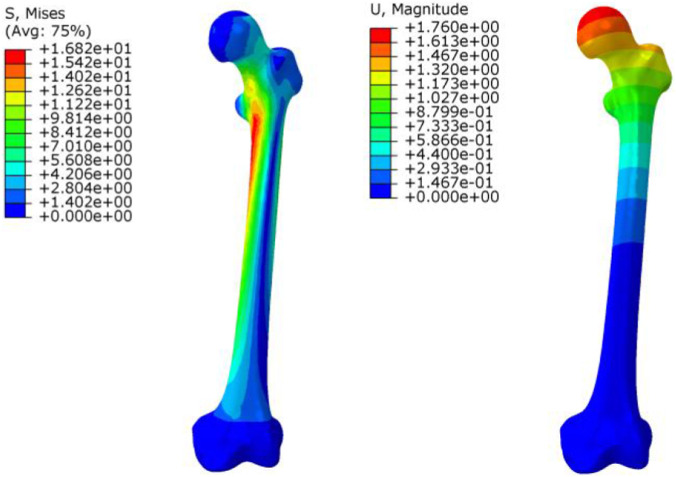
Stress and displacement contour distributions of the intact femur.

To assess the accuracy and biomechanical reliability of the finite element model, validation analyses were performed at both the intact femur level and the fixation model level. First, following commonly adopted methods in previous finite element studies (Keyak et al., 1990; Viceconti et al., 2005), a 700 N axial load was applied to the intact femur model to simulate physiological loading during single-leg stance. The results (Figure 3) showed that the maximum von Mises stress was primarily concentrated in the medial cortical region of the femoral neck, with a peak value of 16.82 MPa. The overall deformation pattern was consistent, and the maximum displacement occurred at the femoral head, with a value of 1.76 mm. The stress distribution pattern indicated that the load was transmitted from the femoral head along the medial cortex of the femoral neck toward the femoral shaft, consistent with the physiological load transfer characteristics of the proximal femur. In addition, both stress and displacement values were comparable to those reported in previous studies (Keyak et al., 1990; Taddei et al., 2004), suggesting that the intact femur model demonstrated reasonable biomechanical consistency. Furthermore, to assess the fixation model, the RN model (Table 3) was analyzed under the same 700 N axial loading condition. The results were compared with those reported in previous finite element and biomechanical studies (Shengyuan, 2022). The stress distribution characteristics and displacement ranges obtained in this study were generally consistent with those reported in the literature, further supporting the reliability of the finite element model for internal fixation analysis.

**TABLE 3:** Comparison results with previous literature.

Parameters	Present study	Tian et al. (2022)
Implant von mises stress (MPa)	149.92	160.39
Femoral displacement (mm)	4.16	4.56

### Stress and displacement distribution

#### Axial loading

**FIGURE 4:**
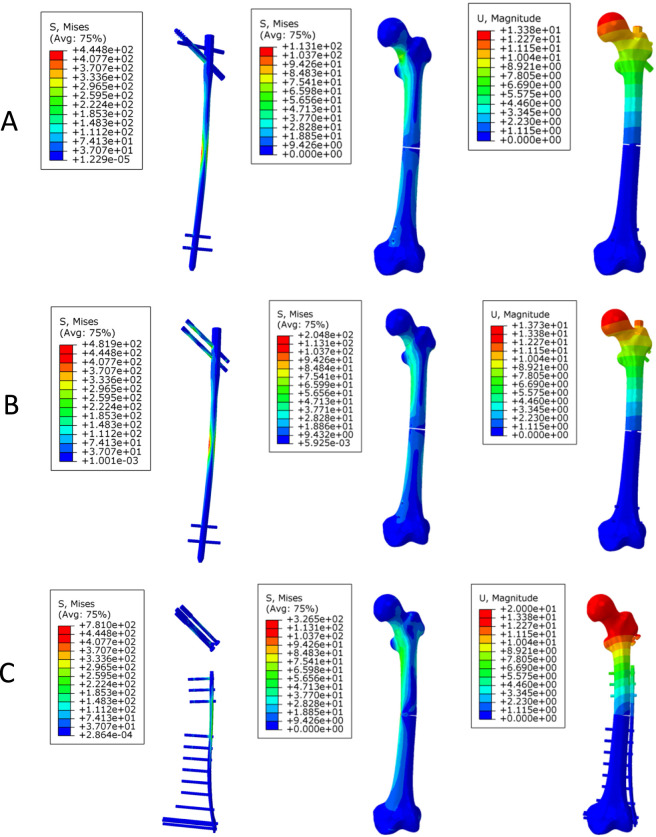
Stress and displacement distribution contour plots of the three models under axial loading. **(A)** long PFBN **(B)** RN **(C)** TCP.

Under axial loading (Figure 4), the PFBN exhibited lower implant stress (444.8 MPa) than RN (481.9 MPa) and TCP (781.0 MPa). A similar trend was observed for femoral stress, with PFBN (113.1 MPa) showing lower values than RN (204.8 MPa) and TCP (326.5 MPa). Displacement followed a comparable pattern: PFBN showed the lowest displacement (13.4 mm), RN exhibited similar values (13.7 mm), and TCP showed higher displacement (20.0 mm).

#### Bending loading

**FIGURE 5:**
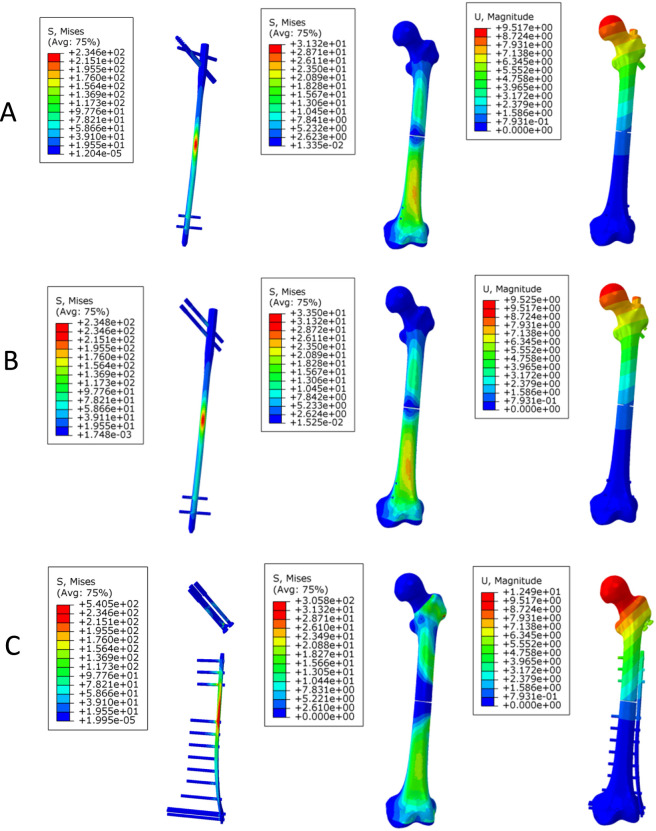
Stress and displacement distribution contour plots of the three models under bending loading. **(A)** long PFBN **(B)** RN **(C)** TCP.

Under bending loading (Figure 5), the implant stress of the PFBN (234.6 MPa) was comparable to that of RN (234.8 MPa), whereas TCP exhibited higher stress (540.5 MPa). Femoral stress in the TCP model (305.8 MPa) was higher than that in PFBN (31.3 MPa) and RN (33.5 MPa). Displacement values were similar between PFBN (9.52 mm) and RN (9.53 mm), whereas TCP exhibited higher displacement (12.49 mm).

#### Torsional loading

**FIGURE 6:**
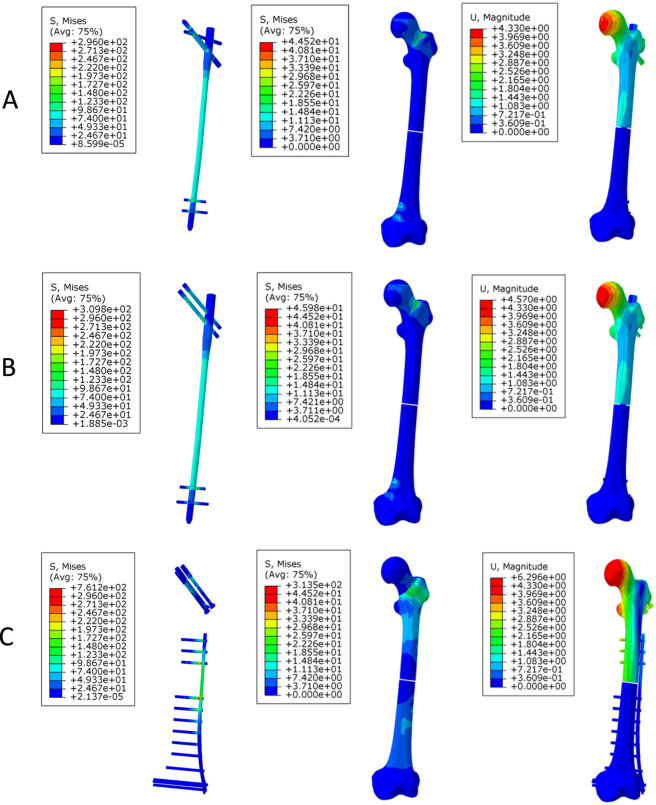
Stress and displacement distribution contour plots of the three models under torsional loading. **(A)** long PFBN **(B)** RN **(C)** TCP.

Under torsional loading (Figure 6), the PFBN exhibited lower implant stress (296.0 MPa) than RN (309.8 MPa) and TCP (761.2 MPa). Femoral stress was higher in the TCP model (313.5 MPa) than in PFBN (44.5 MPa) and RN (46.0 MPa). Displacement showed a similar trend, with PFBN (4.33 mm) lower than RN (4.57 mm) and TCP (6.29 mm).

The biomechanical parameters of the three models under axial, bending, and torsional loading conditions are summarized in Table 4.

**TABLE 4:** Biomechanical evaluation parameters of the three models under axial, bending, and torsional loading.

Load	Parameters	Long PFBN	RN	TCP
Axial	VMS of implant models (MPa)	444.8	481.9	781.0
	VMS of femur models (MPa)	113.1	204.8	326.5
	Displacement (mm)	13.38	13.73	20.00
Bending	VMS of implant models (MPa)	234.6	234.8	540.5
	VMS of femur models (MPa)	31.32	33.50	305.8
	Displacement (mm)	9.517	9.525	12.49
Torsional	VMS of implant models (MPa)	296.0	309.8	761.2
	VMS of femur models (MPa)	44.52	45.98	313.5
	Displacement (mm)	4.330	4.570	6.290

### Tangential micromotion

**FIGURE 7:**
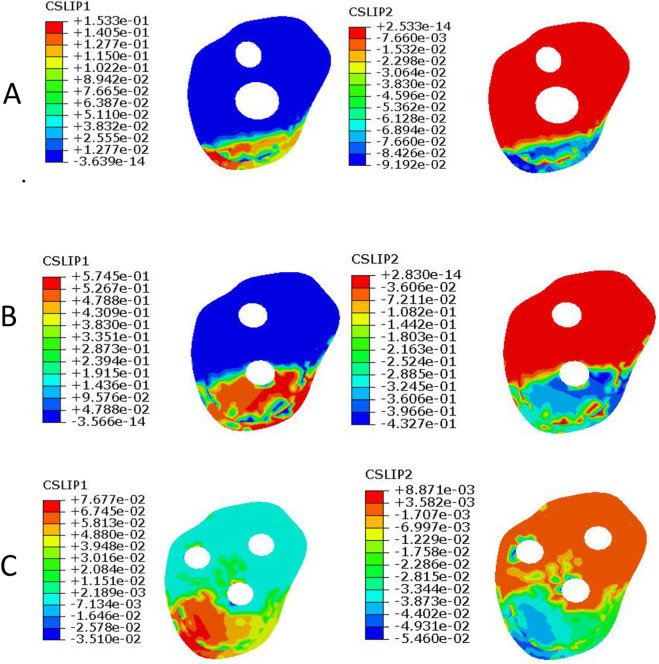
Tangential micromotion at the femoral neck fracture site under axial loading. **(A)** long PFBN, **(B)** RN, **(C)** TCP.

**FIGURE 8:**
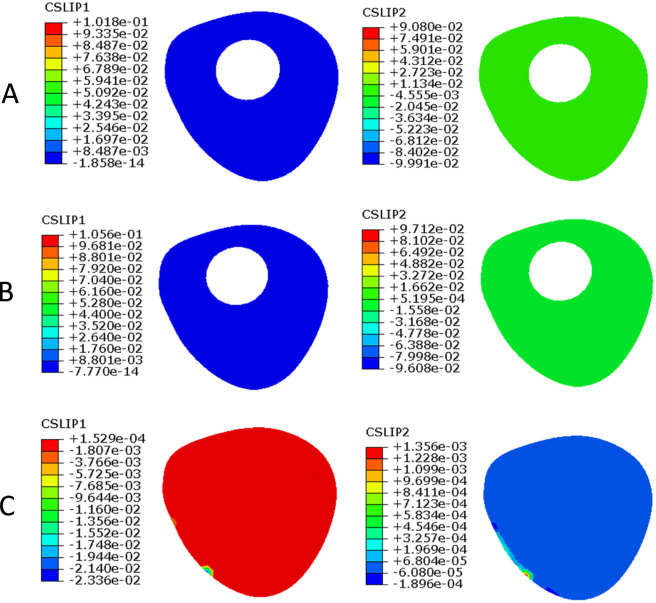
Tangential micromotion at the femoral shaft fracture site under axial loading. **(A)** long PFBN **(B)** RN **(C)** TCP.

CSLIP1 and CSLIP2 represent tangential micromotion of the contact interface in two orthogonal directions and were used to characterize micromotion at the fracture interface. Under axial loading, at the femoral neck fracture interface (Figure 7), the CSLIP1 values for PFBN, RN, and TCP were 0.153 mm, 0.575 mm, and 0.077 mm, respectively, while the corresponding CSLIP2 values were 0.092 mm, 0.433 mm, and 0.055 mm. At the femoral shaft fracture interface (Figure 8), the CSLIP1 values for PFBN, RN, and TCP were 0.102 mm, 0.106 mm, and 0.023 mm, respectively, while the corresponding CSLIP2 values were 0.091 mm, 0.097 mm, and 0.0013 mm, respectively.

## Discussion

This study showed that, compared with two conventional fixation methods, the long PFBN, based on the triangular-supported fixation concept, demonstrated relatively improved stress distribution, displacement control, and fracture-site stability under axial, bending, and torsional loading conditions in the fixation of IFNSF. From a biomechanical perspective, the long PFBN may provide potential advantages in terms of load distribution and structural stability for this type of complex fracture.

The findings of this study suggest that the long PFBN may exhibit several distinct biomechanical characteristics compared with conventional fixation methods. First, in terms of load transfer, the proximal bionic triangular structure combined with distal intramedullary fixation may provide a more continuous load transfer pathway. Load may be transmitted from the femoral head through the proximal triangular structure to the intramedullary nail and subsequently distributed distally along the nail. This configuration may facilitate a more uniform stress distribution and potentially reduce local stress concentration. Second, regarding overall stability, the combined effect of the proximal triangular configuration, intramedullary fixation, and distal locking mechanism may enhance the resistance of the fixation system to axial, bending, and torsional loading. In addition, tangential micromotion at the femoral neck fracture site in the long PFBN model was intermediate between that of the RN and TCP models. Previous studies (Zhao et al., 2025; Ni et al., 2016) have suggested that, under certain conditions, fracture-site micromotion in the range of approximately 0.15 –0.4 mm may be associated with callus formation. However, this range is influenced by multiple factors, including loading conditions, micromotion direction, and the biological environment, and its applicability remains uncertain. In the present study, micromotion in one direction (0.153 mm) fell within this range, whereas that in the other direction (0.092 mm) was slightly below it. These findings may indicate that the long PFBN may limit excessive sliding while maintaining a certain degree of mechanical stimulation. However, whether this biomechanical characteristic translates into a more favorable healing environment remains unclear and requires further experimental and clinical validation. Therefore, the micromotion level observed in the long PFBN model may represent a relatively balanced mechanical environment between fixation stability and mechanical stimulation.

Further analysis of the relationship between structural features and mechanical performance suggests that the design characteristics of the long PFBN may contribute to its observed biomechanical behavior. First, the proximal support screw of the PFBN passes through the tension screw and, together with the proximal nail, forms a triangular stabilizing structure (Zhang Yi-Fan et al., 2024). This triangular configuration is geometrically similar to the distribution of compressive and tensile trabeculae in the Ward’s triangle region of the proximal femur (Ding et al., 2023), which may facilitate a more uniform transfer of load from the femoral head to the femoral shaft. Second, the long PFBN maintains an intramedullary central fixation pattern. Its centralized position may provide mechanical stability while preserving a certain level of physiological stress stimulation, which may help reduce stress shielding effects (Yang et al., 2025). In addition, the threaded locking between the proximal support screw and the main nail may provide angular stability, whereas the limited sliding permitted by the tension screw within the nail channel may contribute to a form of “dynamic stability.” This may help resist excessive shear motion while allowing controlled axial compression under loading conditions (Cheng et al., 2023). It should be noted that these structure–function relationships are primarily inferred from finite element analysis. Their biological implications remain uncertain and require further validation through experimental and clinical studies.

In clinical practice, RN and TCP are commonly used fixation methods for IFNSF. The RN provides intramedullary central fixation and allows simultaneous management of both fractures using a single implant, thereby maintaining continuity of load transfer. In contrast, the TCP strategy employs two fixation systems to stabilize the femoral neck and shaft separately, providing relatively strong local stability. However, both approaches may be associated with certain biomechanical limitations. Proximally, both systems typically adopt parallel screw configurations, which, under complex loading conditions, may be associated with stress concentration at the fixation margins and the screw–bone interface (Cheng et al., 2023), potentially increasing the risk of screw cut-out or loosening. In addition, the parallel configuration lacks a three-dimensional locking effect, which may be associated with complications such as coxa vara, femoral neck shortening (Cheng et al., 2023), and torsional micromotion, potentially influencing the fracture healing environment (Filipov, 2011). Furthermore, in the TCP system, the screws and plate are not integrated into a single construct, which may lead to discontinuous load transfer between components. This may contribute to localized stress concentration within the implant system. Moreover, the rigid fixation provided by the femoral shaft plate may contribute to stress shielding (Olmos et al., 2022), potentially reducing physiological mechanical stimulation at the fracture site and thereby influencing the healing process. In some cases, these factors may be associated with complications such as fixation failure, delayed union, or nonunion.

The findings of this study preliminarily suggest that the long PFBN may exhibit potential biomechanical advantages. For patients with unstable femoral neck fractures combined with comminuted femoral shaft fractures, the anti-rotational capability of the long PFBN may help mitigate certain limitations of conventional reconstruction nails. Compared with combined fixation strategies, its single-implant design may provide a structural basis for reduced surgical invasiveness. However, its minimally invasive characteristics and potential clinical benefits require further validation through well-designed clinical studies. Future research may focus on optimizing the structural parameters of the long PFBN and conducting targeted clinical investigations. In particular, its potential applicability may be further explored in patients with different fracture patterns and varying bone quality.

This study has several limitations that should be acknowledged. First, the finite element model was constructed based on CT data from a single healthy adult male femur, which does not account for variations in bone quality, age-related changes, osteoporosis, sex differences, or fracture complexity in clinical populations. This limitation may affect the generalizability of the findings. Second, soft tissues such as muscles and tendons were not included in the model, although they play an important role in load transmission in the proximal femur. Their exclusion may influence the predicted stress distribution and displacement patterns under physiological conditions. Third, the biomechanical performance of the long PFBN was evaluated only within a deterministic finite element framework, without direct experimental validation. Therefore, the results should be interpreted as comparative biomechanical findings under specific modeling assumptions rather than definitive evidence of clinical superiority. In addition, multiple subject-specific models were not established, which limits the assessment of the robustness of the findings under different conditions. Future studies should incorporate inter-individual variability, more physiologically representative modeling conditions, cadaveric biomechanical experiments, and clinical investigations to further validate and extend the present findings.

## Conclusion

In this finite element study, the long PFBN demonstrated relatively favorable biomechanical performance under axial, bending, and torsional loading conditions. These findings may provide comparative biomechanical insights into the performance of different fixation strategies for this complex fracture pattern.

## Data Availability

The original contributions presented in the study are included in the article/Supplementary Material, further inquiries can be directed to the corresponding authors.
