# Supplementary figures and images for: Finite element analysis of long proximal femoral bionic nail (PFBN) fixation for femoral shaft fractures combined with ipsilateral femoral neck fractures
Source: Front Bioeng Biotechnol. 2026 Apr 22;14:1799331. doi: 10.3389/fbioe.2026.1799331 (PMC13147159; doi:10.3389/fbioe.2026.1799331)

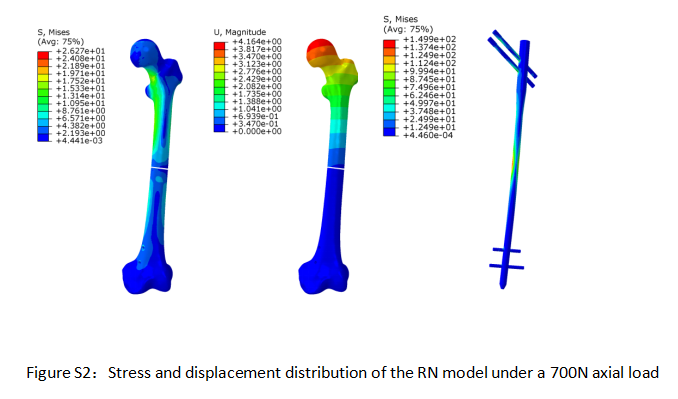

Supplement: Supplementary file 1 [file Image2.png]

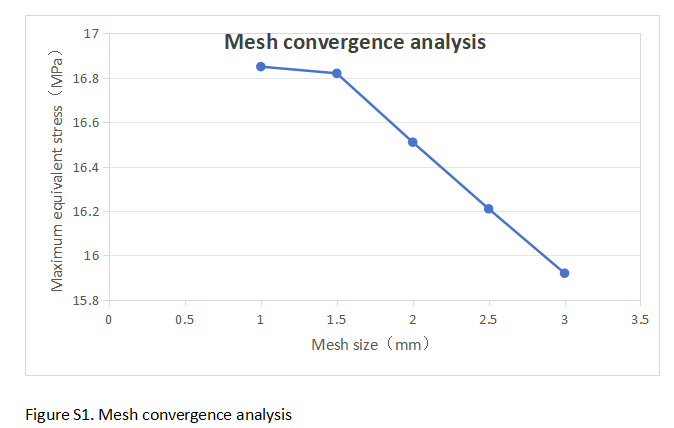

Supplement: Supplementary file 2 [file Image1.png]

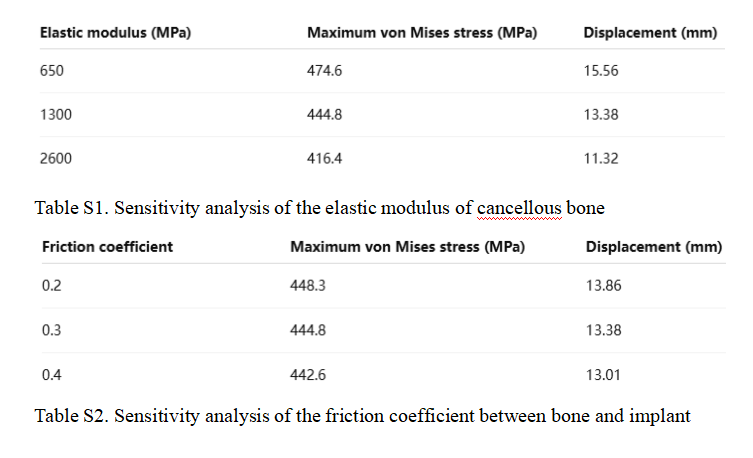

Supplement: Supplementary file 3 [file Image3.png]
